# Engineering Dynamic Hydrophobic Domains in Bioreinforced Ionic Hydrogels for Robust and Transparent Soft Electronics

**DOI:** 10.1021/acs.langmuir.6c01278

**Published:** 2026-06-09

**Authors:** Ijaz Ali, Mansoor Khan, Eliaquim B. P. Sena, André R. Fajardo

**Affiliations:** a Laboratório de Tecnologia e Desenvolvimento de Compósitos e Materiais Poliméricos (LaCoPol), Federal University of Pelotas, Pelotas, RS 96010-900, Brazil; b School of Materials Science and Engineering, 34747 Shanghai University, Shanghai 200444, China

## Abstract

Conductive hydrogels are attractive platforms for soft electronic materials; however, many high-performance systems rely on conductive fillers, complex multinetwork architectures, or multistep processing that compromise scalability and structural clarity. Here, we report a bioreinforced, filler-free ionic hydrogel engineered through micelle-mediated dynamic hydrophobic domains within a hydrogen-bonded polymer network. Stearyl methacrylate is incorporated into a poly­(acrylamide) matrix via SDS-assisted micellization, while gelatin serves as a renewable macromolecular reinforcement, establishing a cooperative network governed by reversible hydrophobic associations and extensive hydrogen bonding. This structure-guided design enables efficient energy dissipation and rapid elastic recovery without permanent structural damage. The optimized hydrogel exhibits ultrahigh stretchability (2420%), enhanced fracture stress (0.31 MPa), high optical transparency (∼85.9%), and low mechanical hysteresis, while maintaining stable ionic conductivity through NaCl incorporation. The dynamic network architecture supports reliable electromechanical response over a wide strain range (0.5–650%), with a maximum gauge factor of 12.08, fast response/recovery times, and excellent cyclic durability. Beyond device-level performance, this work demonstrates how controlled micelle-mediated hydrophobic domain engineering in a bioreinforced polymer matrix can generate mechanically robust, transparent, and conductive soft materials without nanofillers or complex processing. The straightforward one-pot synthesis and use of low-cost components highlight the scalability of this platform for next-generation soft electronic and wearable systems.

## Graphical abstract

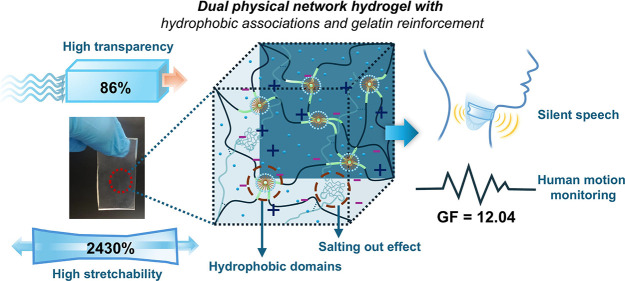

## Introduction

In recent years, conductive hydrogels have attracted significant attention as versatile platforms for flexible and soft electronic materials, including wearable devices,^1,2^ artificial intelligence systems,^3,4^ soft robotics,^5^ biosensing,^6^ biomedical engineering,^7^ information encryption,^8,9^ and human–machine interfaces.^10^ For reliable real-time monitoring under complex deformation, such materials must integrate high stretchability, mechanical robustness, stable electrical or ionic conductivity, and consistent signal response across wide strain ranges.^11,12^ Accordingly, multifunctional conductive hydrogels have emerged as promising material systems for strain sensing, enabling applications such as silent voice recognition,^13^ motion sensing,^14,15^ disease diagnosis,^16^ muscle function assessment,^17^ and gait analysis.^18^ Despite substantial progress in flexibility and sensitivity, persistent trade-offs among toughness, cyclic durability, optical transparency, and sensing stability remain as major challenges, highlighting the need for more rational network design strategies.

Conventional chemically cross-linked hydrogels often exhibit limited fatigue resistance due to permanent covalent junctions that restrict energy dissipation and promote crack propagation under cyclic loading.^15^ Double-network hydrogels partially address this limitation by combining rigid and soft networks; subsequent incorporation of reversible physical interactions further improves fatigue tolerance.^19,20^ However, stress concentration and irreversible damage within sacrificial networks frequently compromise structural integrity under large deformations, leading to gradual mechanical degradation and signal instability. Therefore, developing single-network systems governed predominantly by dynamic physical interactions represents an attractive alternative for constructing mechanically resilient yet structurally recoverable materials.

Dynamic noncovalent interactions, including hydrogen bonding,^21^ electrostatic or ionic interactions,^22,23^ and hydrophobic associations (HpA), have been widely explored to create adaptive hydrogel networks.^24,25^ Among them, HpA-based hydrogels are particularly appealing because hydrophobic domains formed by long alkyl chains act as reversible physical cross-linking nodes. Under mechanical deformation, these domains can dissociate and reform, enabling efficient energy dissipation and autonomous recovery.^26,27^ Hydrogen bonding provides complementary dynamic interactions that enhance elasticity and strain responsiveness.^27,28^ Although combining HpA with additional reinforcing mechanisms such as nanofillers or hierarchical cross-linking improves mechanical strength and sensing performance,^29,30^ many reported systems rely on complex formulations or functional additives that may compromise transparency, structural simplicity, or scalable fabrication.

In parallel, ionic conductive hydrogels have emerged as promising candidates for transparent and compliant soft electronic materials due to their intrinsic transparency and stable ion transport pathways.^31,32^ Their ability to maintain reliable signal output under large deformation and to adhere conformally to soft tissues further supports their application in wearable systems.^33,34^ Nevertheless, achieving a filler-free ionic hydrogel that simultaneously combines high transparency, mechanical robustness, efficient energy dissipation, a wide range of strain sensitivity, and long-term cyclic stability remains challenging. Recent reports continue to highlight limitations such as mechanical fatigue, signal drift, or performance decay under repeated large-strain operation.^35,36^

Integrating HpA with hydrogen bonding within a bioreinforced network offers a promising pathway to overcome these limitations. Gelatin, a biocompatible and biodegradable macromolecule derived from collagen, contains abundant hydroxyl and amino groups capable of forming extensive hydrogen bonds with synthetic polymer chains.^37,38^ In aqueous environments, gelatin aggregation can be influenced by the Hofmeister effect,^39^ potentially stabilizing dynamic network domains. When combined with HpA, such cooperative interactions may create reversible cross-linking nodes capable of dissipating mechanical energy while preserving network integrity. Recent advances in dynamic network engineering, including metal–ligand coordination and hierarchical physical cross-linking, further demonstrate the effectiveness of this strategy for flexible sensing materials.^39,40^

Herein, we engineer a transparent, filler-free ionic hydrogel through micelle-mediated incorporation of hydrophobic domains within a hydrogen-bonded polymer matrix reinforced by gelatin. By synergistically integrating HpA, hydrogen bonding, and ionic conduction within a single physically cross-linked network, we establish a structure–property relationship that enables high stretchability, mechanical resilience, optical transparency, and stable electromechanical response. This work demonstrates how controlled dynamic domain engineering in a bioreinforced polymer system can provide a scalable pathway toward mechanically robust and transparent soft electronic materials.

## Experimental Section

### Materials

Gelatin (Gel, type B from bovine skin, gel strength approximately ∼225 g bloom), stearyl methacrylate (SMA), acrylamide (AAm, ≥99%), sodium chloride (NaCl, 99%), and *N,N,N′,N′*-tetramethylethylenediamine (TEMED, 99%) were purchased from Sigma-Aldrich. Sodium dodecyl sulfate (SDS, ≥90%) was obtained from LabSynth (Diadema, SP, Brazil), while ammonium persulfate (APS, ∼97.5%) was purchased from Êxodo Científica (Brazil). All chemicals were used as received without any chemical treatment.

### Synthesis of Gelatin-Reinforced Hydrophobically Associated Hydrogels

Gelatin-reinforced hydrophobically associated hydrogels were prepared via a one-step free-radical polymerization process. Initially, sodium dodecyl sulfate (SDS, 0.7 g) and NaCl (0.3 g) were dissolved in 9.90 mL of deionized water at 40 °C under magnetic stirring until a clear and homogeneous solution was obtained. Gelatin was then added at a concentration of 4 wt % relative to AAm, and the mixture was continuously stirred at 40 °C for 2 h to ensure complete dissolution. Subsequently, SMA (100 μL) was introduced into the solution, followed by additional stirring at 40 °C for 2 h to promote uniform dispersion of the hydrophobic monomer within the micellar system. Afterward, AAm (2.5 g) was added under continuous stirring, and the mixture was homogenized for 30 min. Once all components were fully dissolved, APS (0.05 g) and TEMED (10 μL) were added sequentially to initiate free-radical polymerization. The resulting precursor solution was immediately poured into a mold and polymerized in an oven at 60 °C for 90 min. After polymerization, the obtained hydrogels were carefully removed from the molds and stored in airtight sample bags at ambient conditions prior to further characterization. After polymerization, the resulting hydrogels were subjected to an exhaustive washing process in distilled water. This washing procedure is essential to ensuring the complete removal of residual monomers (AAm and SMA), surfactants (SDS), and unreacted initiators, thus ensuring the purity and biocompatibility of the material for cutaneous use.

To systematically investigate the effect of gelatin incorporation, a series of gelatin-reinforced hydrogels with varying gelatin contents was prepared following the same synthesis protocol, while keeping all other components (water, SDS, NaCl, SMA, AAm, APS, and TEMED) and processing conditions constant. In this design, gelatin content was the only independent variable, enabling a direct assessment of its role in modulating the network structure, mechanical properties, and sensing performance. The compositions and corresponding sample codes are summarized in Table 1, where the pristine hydrogel without gelatin is denoted as Pure and the gelatin-containing samples are labeled as GT_*x*_, with *x* representing the gelatin content (in g) added to the formulation.

**Table 1:** Composition of the Synthesized Hydrogel Samples and Coding

sample^a^	H_2_O (mL)	SDS (g)	NaCl (g)	SMA (μL)	AAm (g)	gelatin (g)	APS (g)	TEMED (μL)
pure	9.9	0.7	0.3	100	2.5		0.05	10
GT_0.05_	9.9	0.7	0.3	100	2.5	0.05	0.05	10
GT_0.1_	9.9	0.7	0.3	100	2.5	0.10	0.05	10
GT_0.15_	9.9	0.7	0.3	100	2.5	0.15	0.05	10
GT_0.2_	9.9	0.7	0.3	100	2.5	0.20	0.05	10
GT_0.3_	9.9	0.7	0.3	100	2.5	0.30	0.05	10

a All components were kept constant except for the gelatin content.

### Functional, Morphological, and Optical Transmittance Characterization of Hydrogels

Before characterization, the hydrogel samples were freeze-dried at −60 °C for 24 h. Fourier transform infrared (FTIR) spectra were collected using an Affinity-1 spectrometer (Shimadzu, Japan) in the wavenumber range of 4000–500 cm^–1^ with a resolution of 4 cm^–1^. Prior to analysis, the lyophilized samples were finely ground, mixed with potassium bromide (KBr), and compressed into pellets. The surface morphology of the hydrogels was examined by scanning electron microscopy (SEM) using a JEOL JSM-6610LV instrument. Before observation, the freeze-dried samples (with a condensation temperature of −50 °C and a vacuum of 430 μHg for a period of 48 h) were sputter-coated with a thin gold layer to ensure electrical conductivity. Optical transmittance measurements were performed using a UV–vis spectrophotometer (Lambda 25, PerkinElmer, Singapore) in the wavelength range of 400–800 nm under ambient conditions. Hydrogel samples with dimensions of 30 mm × 2.5 mm × 2.5 mm were used for the measurements.

### Mechanical Properties

The mechanical properties of the hydrogels were characterized using a universal testing machine (UTM, MBIO-I 10 kN, Biopdi, Brazil) equipped with a 10 kN load cell. All measurements were performed in tensile mode at room temperature. Rectangular specimens with dimensions of 50 mm in length, 10 mm in width, and 2.5 mm in thickness were subjected to uniaxial tensile and cyclic loading–unloading tests at a constant crosshead speed of 50 mm min^–1^. Herein, the number of replicates was 3. Tensile stress–strain curves were recorded until fracture, and the toughness (work of fracture per unit volume) was calculated by integrating the area under the corresponding stress–strain curve, as described in eq 1. The Young’s modulus was determined from the slope of the tensile stress–strain curve in the strain range up to 50%. Cyclic loading–unloading tests were performed to evaluate the energy dissipation behavior of the hydrogels, which was quantified by calculating the enclosed area of the hysteresis loop according to eq 2.

U=∫σd∈ 1

$$\Delta U=\int_{\mathrm{loading}}\sigma d\epsilon -\int_{\mathrm{unloading}}\sigma d\epsilon$$ 2

### Conductivity and Strain Sensitivity Analyses

The electrical conductivity of the hydrogels was measured using an electrochemical workstation operated in a two-electrode configuration. Electrochemical impedance spectroscopy (EIS) measurements were performed using a potentiostat (CompactStat, Ivium Technologies, The Netherlands) with an applied AC amplitude of 10 mV over a frequency range from 1 MHz to 0.1 Hz. For the measurements, the hydrogel samples were placed in a Teflon cell equipped with stainless-steel electrodes consisting of two circular plates (1 cm diameter, effective area ≈ 0.785 cm^2^) separated by a fixed distance of 0.2 cm (Figure S1). The electrical conductivity (σ) of the hydrogel was calculated according to eq 3:

$$\mathrm{conductivity}\;(\partial )=\frac{l}{RA}$$ 3

where *R* is the measured electrical resistance, *A* is the cross-sectional area of the hydrogel, and *L* is the distance between the electrodes.

The strain sensitivity of the hydrogels was initially evaluated using a custom-built demonstration system, in which the hydrogel was incorporated into a simple electrical circuit consisting of a light-emitting diode (LED) and a DC power source connected in series. Changes in strain-induced resistance were qualitatively monitored through variations in LED brightness. For quantitative analysis, strain-sensing performance was further investigated using an Autolab electrochemical workstation (Metrohm Autolab PGSTAT302N potentiostat/galvanostat, the Netherlands) operated in chronoamperometric mode (current versus time) under a constant applied voltage of 1 V. Based on the recorded current–time response, the real-time electrical resistance was calculated using Ohm’s law. The relative resistance change (Δ*R*/*R*_0_) was then determined according to eq 4:

$$\mathrm{relative}\;\mathrm{resistance}=\frac{R-R_{0}}{R_{0}}$$ 4

where *R*_0_ denotes the initial resistance at zero strain (ε = 0%) and *R* corresponds to the instantaneous resistance under an applied strain. These values were used to calculate the gauge factor (GF) over a strain range of 0–650%.

For epidermal sensing demonstrations, hydrogel samples with the same dimensions as those used in mechanical analysis were fixed at both ends to copper electrodes connected to an electrochemical workstation and attached to different regions of the human body. The resulting resistance signals were recorded to monitor various human motions, including both large-scale joint movements and subtle activities, such as speaking different words.

## Results and Discussion

### Synthesis and Structural Design of P­(SMA-*co*-AAm) and P­(SMA-*co*-AAm)/Gelatin Hydrogels

Recent studies have demonstrated that HpA-based hydrogels prepared via micellar-free-radical copolymerization provide an effective framework for constructing stretchable and fatigue-resistant soft materials. This strategy relies on micellar polymerization, a well-established approach that enables the incorporation of hydrophobic monomers into aqueous polymer networks through surfactant-mediated solubilization, thereby preventing macroscopic phase separation. Within this framework, SDS self-assembles into micelles that act as nanoscopic solubilization domains for hydrophobic monomers, such as stearyl methacrylate (SMA). These micelle-stabilized hydrophobic domains are subsequently embedded within the polymer network, where they function as dynamic physical cross-linking nodes.^41^ These domains reversibly dissociate and reassociate under mechanical deformation, enabling efficient energy dissipation and structural recovery, a mechanism widely recognized as a key design principle for resilient hydrogel networks.^27^ Although similar HpA architectures have been reported to enhance deformation tolerance and sensing functionality, limitations related to network stability, sensitivity retention, or optical transparency remain, particularly when additional reinforcing components are incorporated.^29,42^

Upon initiation of free-radical polymerization, the SDS/SMA micellar domains become embedded within the growing poly­(acrylamide) matrix, generating a physically cross-linked network in which hydrophobic domains serve as reversible junctions. This micelle-mediated architecture, as supported by the mechanical and structural analyses presented below, minimizes irreversible bonds typically observed in chemically cross-linked or classical double-network hydrogels, thereby promoting rapid autonomous recovery after strain release. While hierarchical structuring and nanoreinforcement strategies have been employed to further enhance mechanical performance, such approaches often increase compositional complexity or compromise optical clarity, which is essential for transparent soft electronic materials.^30^

To reinforce the HpA network while preserving structural simplicity, gelatin was incorporated as a multifunctional bioderived component. Gelatin contains abundant amide (−NH) and carbonyl (C=O) groups along its backbone, as well as polar side-chain functionalities (−OH, −COOH, −NH_2_), enabling extensive hydrogen bonding interactions with P­(SMA-*co*-AAm) chains. These reversible hydrogen bonds operate cooperatively with HpA, increasing network cohesion while maintaining elasticity and recoverability. Although related reinforcement strategies based on dynamic hydrogen bonding or metal–ligand coordination have been reported,^43^ such systems frequently rely on complex multicomponent designs that may limit scalability or long-term structural stability. In contrast, gelatin provides a simple and biocompatible reinforcing pathway without introducing optical scattering centers.

In addition to hydrogen bonding, Na^+^ and Cl^–^ ions, proceeding from gelatin-associated salts and added NaCl, modulate intermolecular interactions through a salting-out effect.^44^ This phenomenon enhances hydrophobic aggregation and stabilizes micellar domains, thereby strengthening HpA-mediated junctions under large deformation. Importantly, the combined interactions (HpA, hydrogen bonding, and ionic effects) are reversible under ambient conditions. During mechanical loading, these dynamic bonds transiently dissociate to dissipate energy and subsequently reform upon stress release, enabling repeated deformation without permanent structural damage.^45^ Such dynamic behavior is critical for strain sensors operating under cyclic and multidirectional deformations. Comparable ionic hydrogel systems have been reported; however, performance degradation or limited operational strain ranges remain common.^46,19^

Furthermore, NaCl incorporation establishes continuous ionic conduction pathways within the transparent network, enabling electromechanical signal transduction without the use of conductive fillers. Unlike hybrid conductive hydrogels that depend on nanoparticles or carbon-based additives, which may impair transparency or mechanical compliance, the present design integrates ionic conductivity, stretchability, and self-recovery within a single physically cross-linked architecture. Recent literature highlights persistent trade-offs among sensitivity, durability, and transparency in conductive hydrogel sensors.^20,35,47^ By engineering cooperative hydrophobic and hydrogen-bonded domains within a bioreinforced matrix, the present system establishes a structurally coherent network that balances mechanical robustness, optical clarity, and stable ionic response. A schematic representation of the proposed network architecture is shown in Figure 1, illustrating the synergistic contributions of HpA domains, hydrogen-bonded junctions, and ionic conduction pathways. These design principles are further supported by the spectroscopic, morphological, and mechanical results discussed in the following sections, which collectively confirm the formation of a dynamically cross-linked and structurally coherent network.

**Figure 1:**
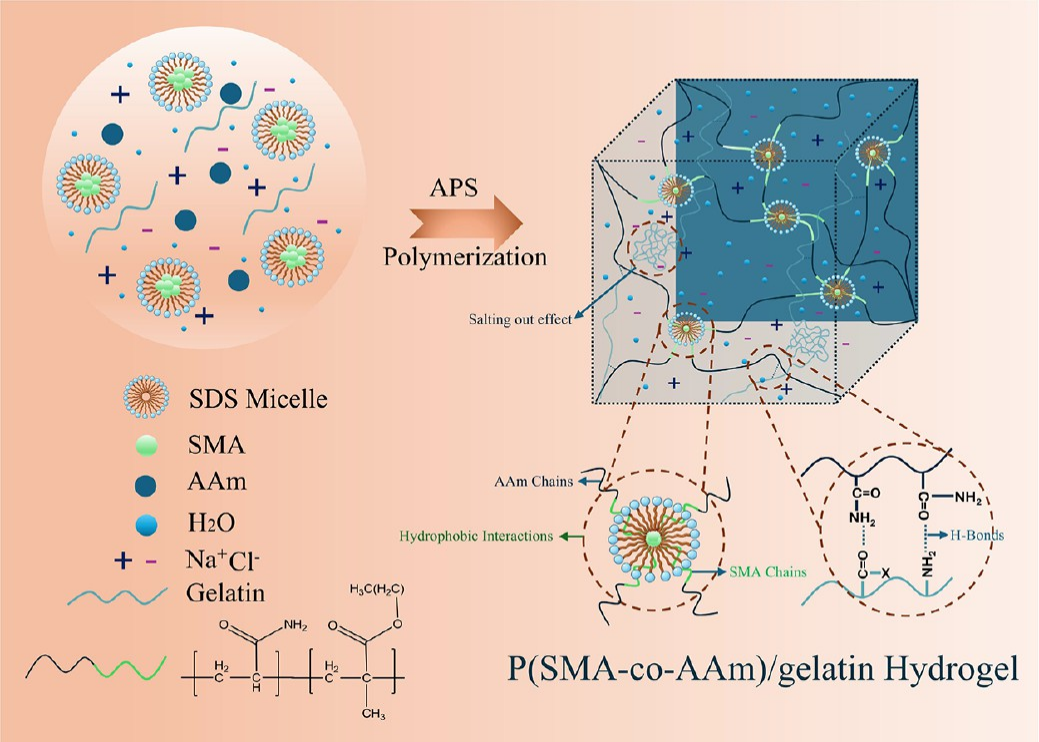
Schematic illustration of the synthetic route and the major interactions involved in the P­(SMA-*co*-AAm)/gelatin hydrogel formation.

To experimentally validate the proposed network design, FTIR spectroscopy was employed to investigate the molecular interactions contributing to the formation of the physically cross-linked hydrogel networks. As shown in Figure 2a, all samples display characteristic absorption bands at approximately 2851 and 2924 cm^–1^, corresponding to the symmetric and asymmetric stretching vibrations of C–H bonds in long alkyl chains. These signals are consistent with the incorporation of SMA within SDS-stabilized micellar domains, supporting the presence of hydrophobic segments embedded in the polymer matrix.^48,49^ Similar spectral features have been reported in hydrophobically associated hydrogels prepared via micellar copolymerization.^29^ Although FTIR does not directly resolve micellar structure, the persistence of these alkyl stretching bands indicates the retention of hydrophobic domains within the cross-linked network.

**Figure 2:**
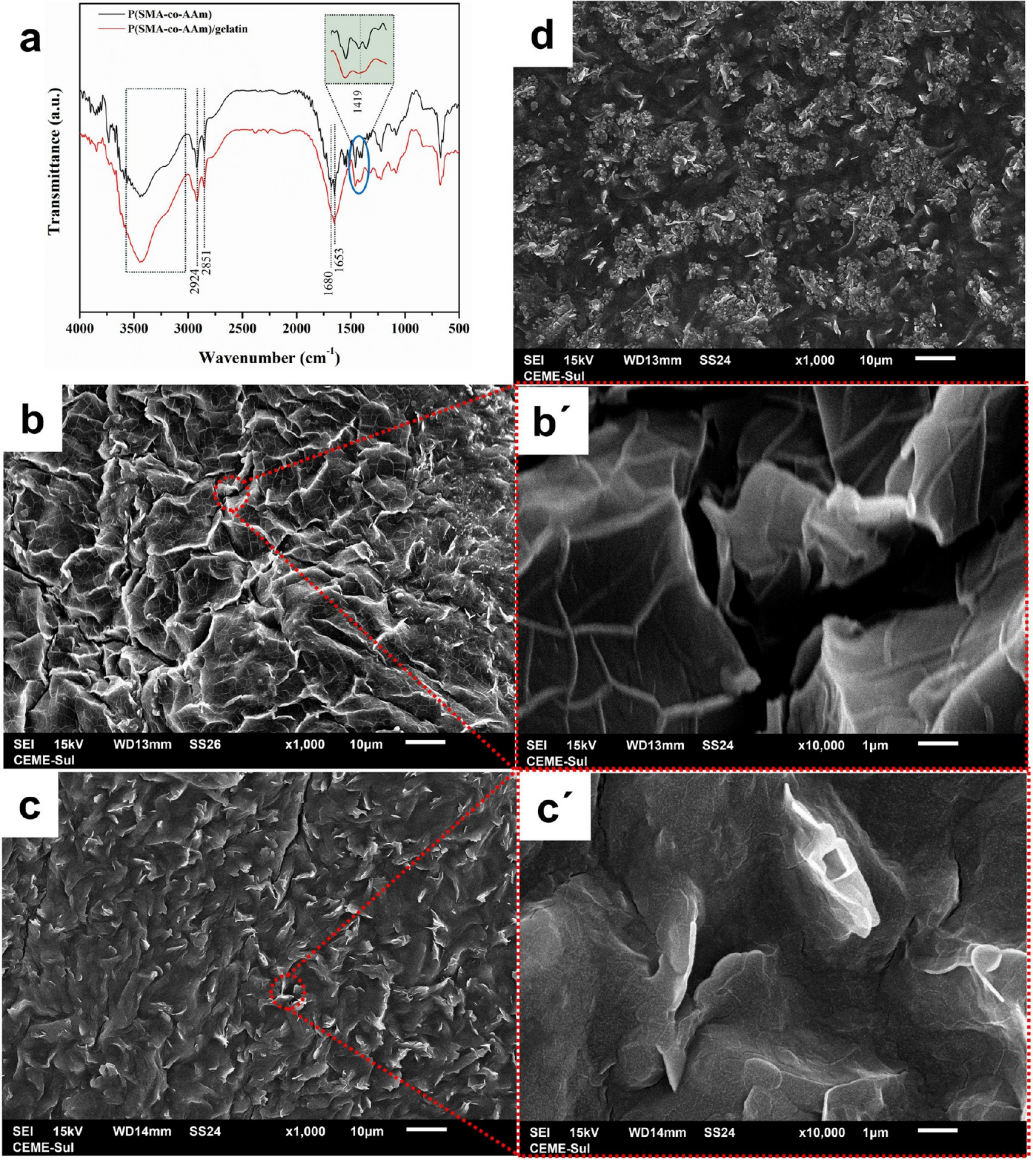
FTIR analysis of Pure and GT_0.15_ hydrogels. SEM images of (b) the pure hydrogel sample and (b′) a magnified view of the selected region of pure hydrogel; (c) GT_0.15_ and (c′) a magnified view of the selected area of GT_0.15_; and (d) GT_0.2_.

A broadband in the 3000–3700 cm^–1^ region is observed for all formulations, arising from overlapping N–H and O–H stretching vibrations associated with amide and hydroxyl groups, as well as intra- and intermolecular hydrogen bonding interactions.^50^ Upon gelatin incorporation, the C=O stretching band shifts from 1680 to 1653 cm^–1^, indicating increased hydrogen bonding interactions between gelatin chains and the P­(SMA-*co*-AAm) backbone.^51^ This red shift suggests enhanced intermolecular association and increased network cohesion due to cooperative hydrogen bonding. Such hydrogen-bond-mediated reinforcement has been reported to strengthen physically cross-linked hydrogels, although often in more complex multicomponent systems.^40,43^ Additionally, the band near 1400 cm^–1^, assigned to CH_*x*_ bending and stretching vibrations, shifts and partially overlaps at approximately 1419 cm^–1^ after gelatin incorporation. This spectral evolution is consistent with modified chain packing and strengthened hydrophobic interactions, potentially influenced by salting-out effects associated with gelatin-bound ions.^52^ The combined spectral changes (i.e., preservation of alkyl stretching bands, C=O red shift, and CH_*x*_ band evolution) support the coexistence of HpA and hydrogen bonding within the network. Together, these reversible interactions establish a dual physically cross-linked architecture capable of cooperative energy dissipation and structural recovery under deformation. Such synergistic dynamic interactions are increasingly recognized as important contributors to mechanical resilience and sensing stability in physically cross-linked hydrogel systems.^19^

The morphological characteristics of the hydrogels were examined by SEM, as shown in Figure 2b–d for the Pure, GT_0.15_, and GT_0.2_ samples, respectively. The Pure hydrogel displays a relatively rough and irregular surface with visible microcracks. These features can be partially attributed to shrinkage-induced stress during sample drying prior to SEM observation; however, the loosely interconnected porous structure also suggests limited network compactness and weaker physical cross-linking density. In contrast, upon incorporation of 0.15 g gelatin (GT_0.15_), the hydrogel exhibits a more homogeneous and interconnected porous morphology with a comparatively smoother surface. The enhanced structural uniformity is consistent with increased intermolecular interactions between gelatin and P­(SMA-*co*-AAm) chains through hydrogen bonding, which likely improves network cohesion. A more organized porous architecture may facilitate continuous ion transport pathways while maintaining mechanical flexibility.

Further increasing the gelatin content to 0.2 g (GT_0.2_) results in a less uniform and partially aggregated morphology. The appearance of structural heterogeneity suggests that excessive gelatin disrupts the balance between micellar HpA and polymer chain organization during network formation. Similar reinforcement-homogeneity trade-offs have been reported in gelatin- or biopolymer-modified hydrogels, where excessive additive incorporation leads to phase irregularities and compromised structural stability.^35,47^ Consistent with this observation, the formulation containing 0.3 g of gelatin (not shown) exhibited pronounced structural nonuniformity and reduced mechanical integrity and was therefore excluded from further study.

Collectively, the SEM observations, together with the FTIR results, indicate that an optimal gelatin concentration promotes a structurally coherent dual physically cross-linked network, whereas excessive incorporation induces heterogeneity that may compromise mechanical and optical performance.

### Mechanical Properties of P­(SMA-*co*-AAm)/Gelatin Hydrogel

High stretchability, mechanical resilience, and durability under cyclic deformation are essential for soft electronic materials intended to emulate the compliance of human skin. To qualitatively evaluate deformability, a series of macroscopic demonstrations were performed. As shown in Figure 3a–c, the GT_0.15_ hydrogel sustains large tensile deformation and complex mechanical manipulations, including bending, twisting, and knotting, without visible fracture. The material can be elongated to approximately five times its initial length while maintaining structural integrity, indicating effective energy dissipation through reversible physical cross-linking.

**Figure 3:**
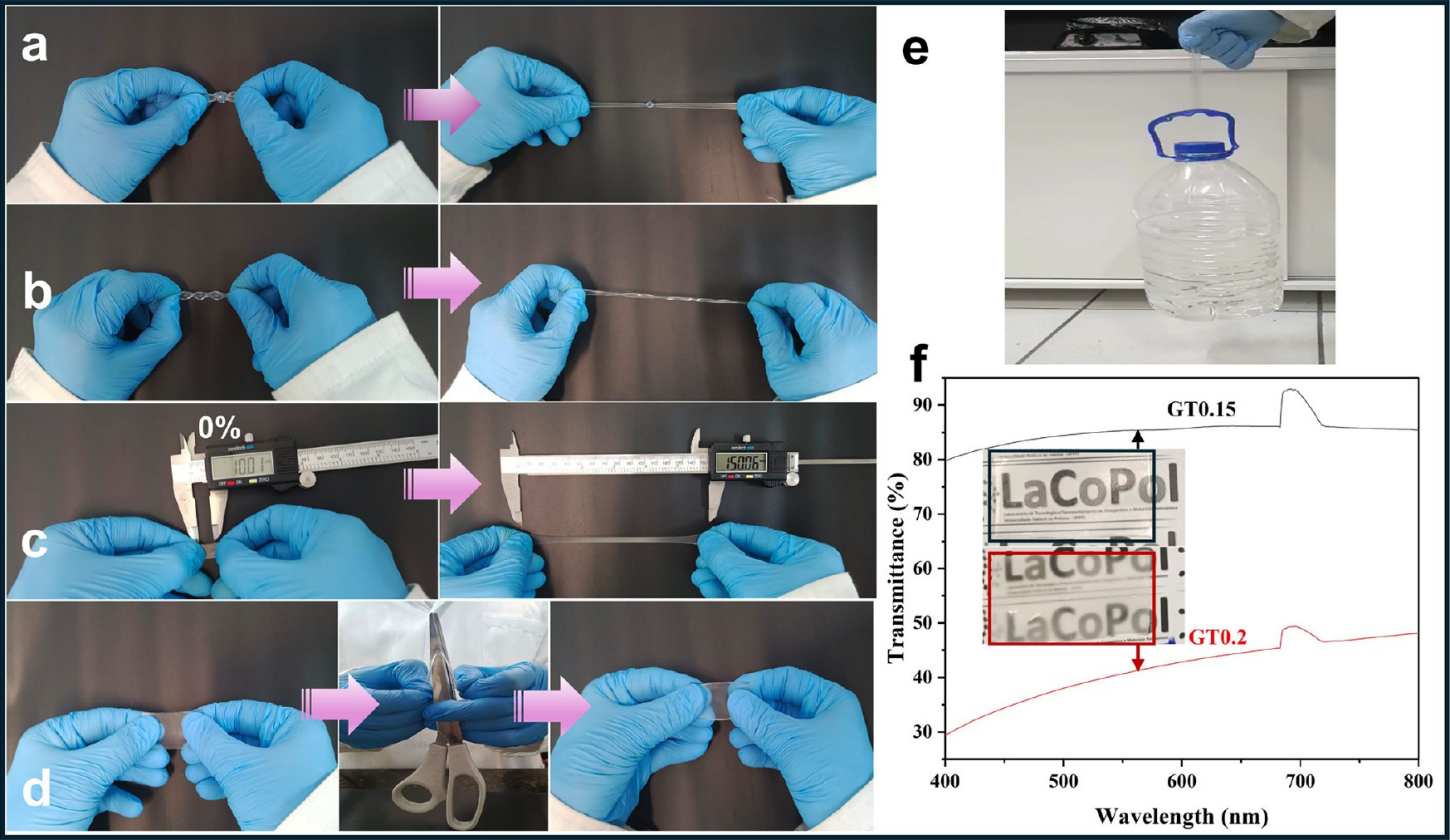
Pictures for the demonstration of the mechanical behavior GT_0.15_ sample: (a, b) curling and knotting tests show its high flexibility and stretchability. (c) Stretchability measurement using a vernier caliper. (d) Hydrogel puncturing resistance test. (f) Load-bearing test showing the hydrogel’s capability to sustain more than 2 kg. (e) Transparency demonstration of the GT_0.15_ and GT_0.2_ samples.

The resistance to localized stress was further evaluated through puncture and load-bearing tests. As shown in Figure 3d, the GT_0.15_ hydrogel tolerates concentrated stress applied by scissor tips without catastrophic failure, suggesting an efficient redistribution of stress through dynamic network junctions. Additionally, the hydrogel supports loads exceeding 2 kg without observable macroscopic damage (Figure 3e). Such behavior reflects enhanced network cohesion compared with many physically cross-linked conductive hydrogels reported in the literature, where high stretchability is often accompanied by limited resistance to stress concentration.^29,42^

Beyond mechanical compliance, optical transparency is a critical parameter for transparent soft electronic materials, as it reflects internal structural homogeneity and minimal light scattering. The transmittance of GT_0.15_ and GT_0.2_ hydrogels (400–800 nm) are shown in Figure 3f. The GT_0.15_ hydrogel exhibits high optical transparency, reaching approximately 85.9% across the visible range. This high transmittance suggests a uniform internal network with limited phase separation. Comparable or higher transparency than many filler-containing conductive hydrogels has been reported only in systems with carefully controlled microstructures.^19,46^

In contrast, the GT_0.2_ hydrogel displays a reduced transmittance of approximately 50%, indicating an increased light scattering due to structural heterogeneity and partial aggregation. This trend aligns with the SEM observations and supports the existence of an optimal gelatin concentration. Similar transparency-reinforcement trade-offs have been reported in related systems, where excessive biopolymer incorporation disrupts network uniformity and compromises optical clarity.^47^ On the basis of these results, it can be suggested that the GT_0.15_ formulation achieves a balanced combination of stretchability, resistance to stress concentration, and optical transparency. The cooperative interactions among HpA, hydrogen bonding, and ionic effects provide a dynamic network capable of dissipating mechanical energy while maintaining the structural coherence. This structural balance establishes the mechanical and optical foundation necessary for a stable electromechanical performance under repeated and complex deformation.

To quantitatively evaluate the effect of gelatin incorporation on the mechanical behavior of the hydrophobically associated hydrogel, uniaxial tensile and cyclic loading–unloading tests were performed. The corresponding stress–strain curves are presented in Figure 4a–d. Key mechanical parameters (namely, fracture stress, fracture strain, toughness, fracture energy density, and Young’s modulus) were extracted to assess the structural resilience and deformation tolerance of the network.

**Figure 4:**
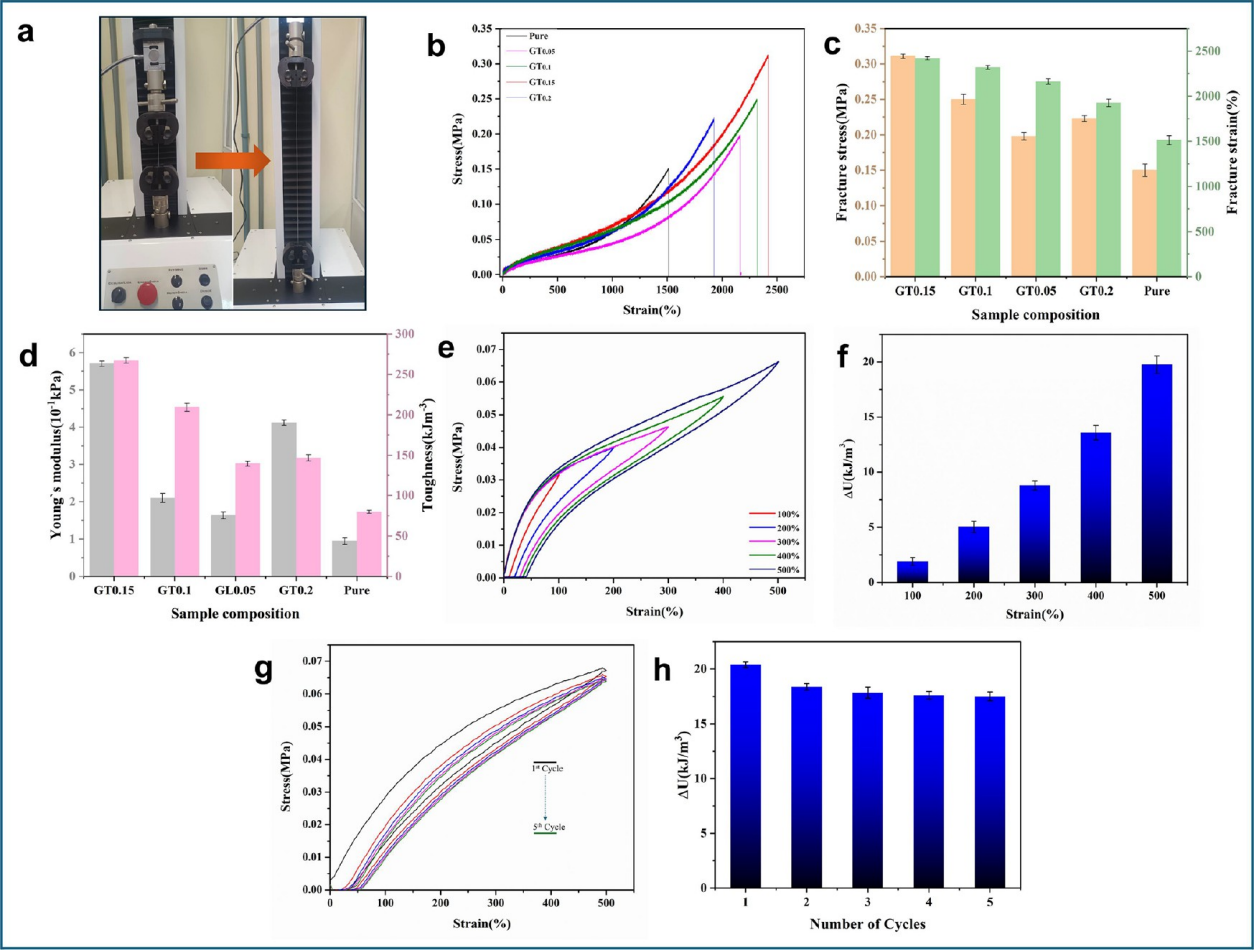
(a) Universal testing machine setup used for tensile and cyclic mechanical testing of the hydrogels before and after deformation. (b) Representative tensile stress–strain curves of Pure and P­(SMA-*co*-AAm)/gelatin hydrogels with different gelatin contents. (c, d) Tensile-derived mechanical parameters, including fracture stress, fracture strain, toughness (stored energy), and Young’s modulus. (e) Cyclic loading–unloading curves at five different applied strains, illustrating reversible deformation behavior of GT_0.15_. (f) Dissipated energy as a function of applied strain, highlighting strain-dependent energy dissipation. (g) Multiple cyclic loading–unloading tests were performed at a constant strain of 500%, demonstrating mechanical durability under repeated deformation of GT_0.15_. (h) Dissipated energy as a function of cycle number, evidencing stable energy dissipation and fatigue resistance.

The pure hydrogel exhibits a fracture stress of 0.15 MPa and a fracture strain of 1512%, consistent with typical hydrophobically associated PAAm-based systems. Upon gelatin incorporation, both fracture stress and fracture strain increase progressively up to an optimal concentration. The GT_0.15_ hydrogel reached a maximum fracture stress of 0.31 MPa and a fracture strain of 2420%. Relative to the Pure sample, fracture stress increases by approximately 32, 66.7, and 107.3% for GT_0.05_, GT_0.1_, and GT_0.15_, respectively. Notably, the concurrent enhancement of strength and extensibility indicates improved load transfer and delayed crack propagation within the physically cross-linked network.

This mechanical reinforcement can be rationalized by the cooperative contributions of multiple reversible interactions. Hydrogen bonding between gelatin and P­(SMA-*co*-AAm) chains likely increases the effective density of physical junctions, while ionic effects associated with gelatin and NaCl may enhance hydrophobic aggregation through salting-out phenomena, stabilizing micellar domains. These dynamic junctions can reversibly dissociate under strain, enabling energy dissipation and stress redistribution. Similar dynamic reinforcement strategies based on hydrogen bonding or HpA have been reported to improve either toughness or stretchability; however, achieving a simultaneous and significant enhancement of both parameters within a single physically cross-linked network remains challenging.^42,46^

Further increasing the gelatin content to 0.2 g results in reduced fracture strain (1925%) and fracture stress (0.223 MPa). This decline suggests that excessive gelatin perturbs the optimized balance between micellar HpA and polymer chain organization during network formation. Previous studies have shown that high gelatin content may interfere with free-radical polymerization, leading to shorter or more disordered chains.^53^ In addition, gelatin–SDS interactions can generate complex micellar aggregates due to their amphiphilic character.^54,55^ At elevated gelatin concentrations, stronger gelatin-SDS binding^56^ may destabilize micellar structures and reduce the availability of effective hydrophobic domains for SMA incorporation. Consequently, the density and integrity of hydrophobic cross-linking points may decrease, leading to diminished mechanical performance. This interpretation aligns with the SEM and optical transmittance results, which revealed increased structural heterogeneity at higher gelatin contents. The GT_0.3_ hydrogel exhibited pronounced nonuniformity and insufficient mechanical integrity and was therefore excluded from further analysis. Similar reinforcement-disruption trade-offs have been reported in conductive hydrogels incorporating excessive secondary phases or biopolymers.^35,47^

A comparable trend is observed for toughness and Young’s modulus (Figure 4d). The Pure hydrogel displays a toughness of 79.7 kJ m^–3^ and a Young’s modulus of 0.094 kPa. Incorporation of 0.15 g gelatin increases toughness to 267.1 kJ m^–3^ and Young’s modulus to 0.6 kPa, indicating enhanced energy storage capacity and improved resistance to deformation. At 0.2 g gelatin, toughness decreases to 146.8 kJ m^–3^ and Young’s modulus to 0.223 kPa, further confirming the existence of an optimal reinforcement level. Together, these results demonstrate that precise control of gelatin content governs the balance between network reinforcement and structural uniformity. The GT_0.15_ hydrogel achieves a mechanically robust yet highly extensible architecture through cooperative dynamic interactions, establishing a structurally optimized single-network system suitable for repeated large-strain deformation.

Cyclic loading–unloading tests were performed at different maximum strain levels to investigate the toughening and energy dissipation behavior of the GT_0.15_ hydrogel (Figure 4e). The dissipated energy increases progressively with increasing applied strain from 100 to 500% (Figure 4f), reaching approximately 1.91 kJ m^–3^ at 100% strain and 19.76 kJ m^–3^ at 500% strain. This monotonic increase suggests strain-activated dissociation of reversible physical cross-linking domains, consistent with progressive disruption and reorganization of HpA and hydrogen bonds during deformation.

The magnitude of dissipated energy is lower than that reported for many highly toughened hydrogels designed for extreme mechanical robustness, which often rely on sacrificial bond rupture mechanisms and can exhibit values exceeding 30–50 kJ m^–3^ at comparable strains. However, for strain-sensing applications, moderate and controlled energy dissipation can be advantageous. Excessive internal damage accumulation may induce mechanical hysteresis, delayed recovery, and signal instability during cyclic operation.^20^ Conversely, the present system displays a deformation mode dominated by reversible, nondestructive rearrangement of dynamic interactions, favoring elastic recovery and signal reproducibility.

To further evaluate fatigue resistance, continuous cyclic loading–unloading tests were conducted at 500% strain without resting intervals (Figure 4g). The dissipated energy decreases slightly from 20.39 kJ m^–3^ in the first cycle to 18.37 kJ m^–3^ in the second cycle, followed by stabilization at approximately 17.84 and 17.60 kJ m^–3^ in the third and fourth cycles (Figure 4h). No abrupt decay in energy dissipation or structural failure is observed within five consecutive cycles, indicating a stable mechanical performance under repeated large-strain deformation. The slight decrease between the first and second cycles likely reflects initial structural rearrangement or minor stress relaxation after which the network reaches a mechanically stable state.

The observed fatigue resistance can be attributed to the reversible nature of HpA associations and hydrogen bonding interactions. Under applied strain, these dynamic junctions partially dissociate, dissipating mechanical energy while preserving the integrity of the polymer backbone. Upon unloading, the rapid reformation of these interactions restores the network structure without requiring external stimuli. Although similar recovery mechanisms have been reported in other physically cross-linked hydrogels, many systems exhibit gradual mechanical degradation under high-strain cycling.^40,47^ Chemically cross-linked hydrogels often display limited extensibility and irreversible damage due to permanent covalent bond rupture, restricting their applicability in flexible electronic devices subjected to repeated deformation.

Importantly, the relatively low dissipated energy at 100% strain (∼1.91 kJ m^–3^) indicates that deformation within moderate strain regimes is predominantly elastic. Considering that typical epidermal strains during daily human motion are generally below ∼75%, this mechanical response is particularly relevant for wearable sensing applications. The combination of moderate strain-dependent energy dissipation, rapid elastic recovery, and stable cycling behavior supports the suitability of the GT_0.15_ hydrogel for long-term strain sensing under both subtle and large-amplitude deformations.

### Conductivity and Sensitivity of P­(SMA-*co*-AAm)/Gelatin Hydrogel

Electrochemical impedance spectroscopy (EIS) was employed to evaluate the ionic conductivity of the synthesized hydrogels, as this technique enables a reliable and nondestructive assessment of bulk charge transport in soft materials. The bulk resistance was extracted from the high-frequency intercept of the Nyquist plots, and the conductivity values were calculated accordingly. The presence of NaCl provides mobile Na^+^ and Cl^–^ ions, while the hydrated polymer network offers continuous aqueous pathways that facilitate ion migration, resulting in stable ionic conduction.

The pure hydrogel exhibited an ionic conductivity of 0.0127 S m^–1^. With increasing gelatin content, conductivity increased moderately, reaching 0.042 S m^–1^ for the GT_0.15_ hydrogel (Figures S2 and S3). Since all formulations contained identical NaCl concentrations, the observed variations are attributed primarily to differences in microstructural organization rather than to ionic content. As suggested by SEM analysis, the optimized gelatin content promotes a more homogeneous and interconnected network, which favors efficient ion transport. Importantly, the enhancement in conductivity occurs concurrently with improved mechanical robustness, indicating that reinforcement of the hydrophobically associated network does not compromise the ionic mobility.

To preliminarily demonstrate strain responsiveness, a simple LED-based circuit was assembled (Video S1 and Figure S4). Progressive stretching resulted in gradual dimming of the LED, indicating an increase in electrical resistance. Upon release, the LED rapidly recovered its initial brightness. This reversible behavior reflects deformation-induced changes in the geometry of ionic conduction pathways, including elongation of the network and reduction in effective cross-sectional area, followed by structural recovery upon unloading. For quantitative analysis, strain-sensing behavior was investigated via chronoamperometric measurements under constant applied voltage. As shown in Figure 5a,b, the GT_0.15_ hydrogel exhibits a clear and reproducible increase in relative resistance (Δ*R*/*R*_0_) across a broad strain range, enabling detection of both small deformations (0.5%) and large strains up to 400%. The monotonic resistance increase with strain is consistent with geometric deformation effects and strain-induced reconfiguration of ionic transport pathways. Similar sensing mechanisms have been reported in ionic conductive hydrogels; however, maintaining signal stability at high-strain levels remains challenging in many systems.^19,47^

**Figure 5:**
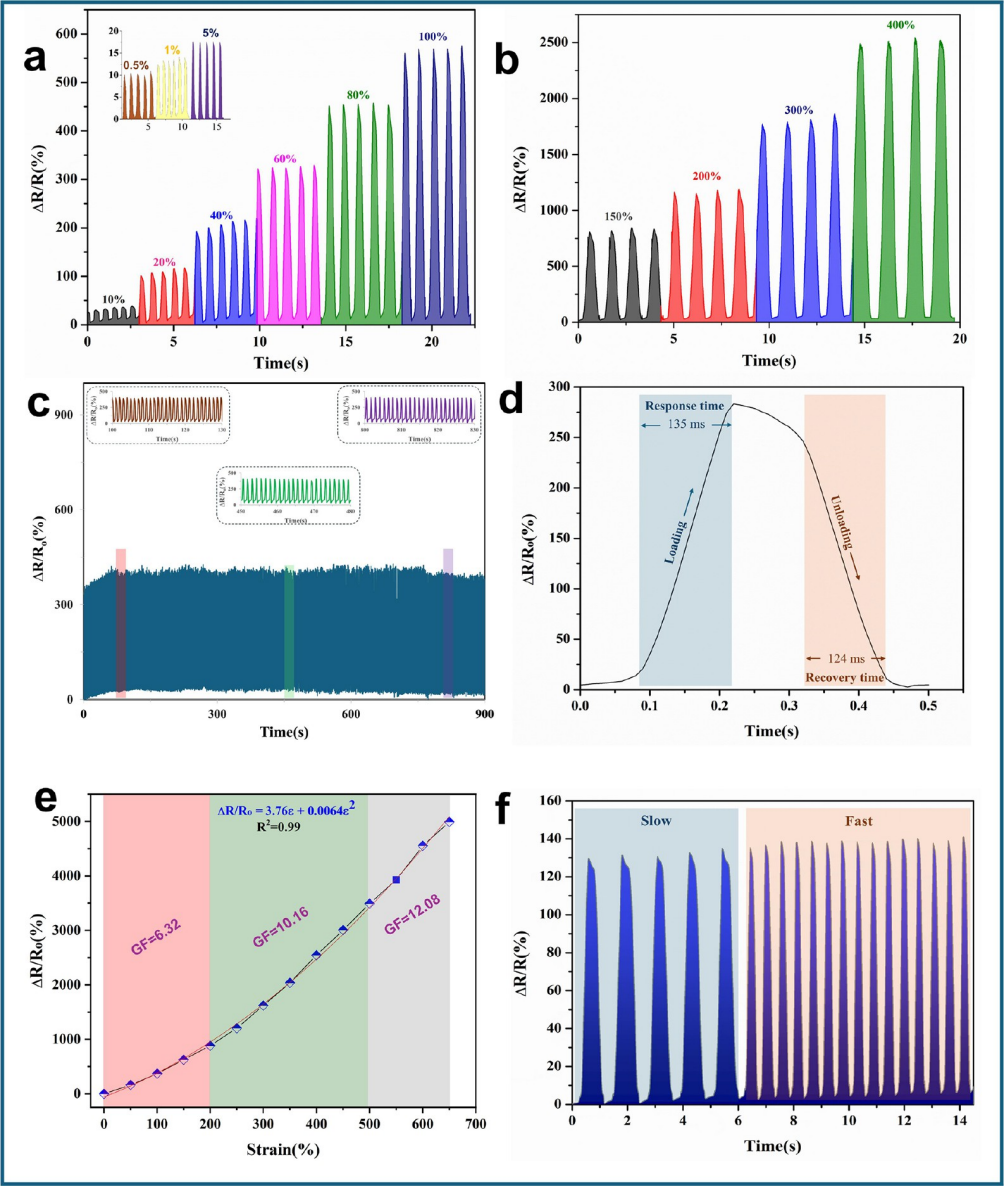
Demonstration of the strain-sensing performance of the GT_0.15_ hydrogel. (a, b) Relative resistance response under various small and large applied strains, illustrating sensitivity across a wide deformation range (the inset in Figure 5a shows a magnified view of the stress–strain response at low-strain range, <10%). (c) Cyclic stability of the hydrogel under repeated loading–unloading at fixed strain, demonstrating signal reproducibility and durability. (d) Response and recovery times of the hydrogel during dynamic deformation. (e) Determination of the GF over a broad strain range of 0–650%, highlighting the multiregime linear sensing behavior. (f) Relative resistance response under slow and fast deformation rates, evaluating the effect of strain rate on sensing performance.

Cyclic stability and signal reproducibility are critical parameters for wearable strain sensors that are subjected to repeated mechanical deformation. To assess fatigue resistance, the hydrogel was subjected to more than 1000 continuous loading–unloading cycles within 900 s at 200% strain (Figure 5c). No noticeable signal drift, degradation, or mechanical failure was observed throughout the cycling process, indicating stable electromechanical coupling under repeated deformation. The minimal variation in the resistance response suggests that the reversible HpA and hydrogen bonds effectively prevent cumulative structural damage during cyclic loading. Compared with many reported hydrogel-based strain sensors that exhibit gradual signal attenuation or mechanical degradation under prolonged cycling, the present system demonstrates reliable durability under relatively large deformation.^35,40^

The dynamic sensing capability of the hydrogel was further evaluated by measuring the response and recovery times. As shown in Figure 5d, the GT_0.15_ hydrogel exhibits a response time of 135 ms and a recovery time of 124 ms, reflecting the rapid reconfiguration of ionic conduction pathways upon deformation and relaxation. Such response characteristics are suitable for the real-time monitoring of human motion, including both subtle physiological signals and large-scale joint movements. These values are comparable to those reported for high-performance ionic conductive hydrogels that combine stretchability with mechanical robustness.^57,43^

The gauge factor (GF), defined as the ratio between the relative resistance change and the applied strain $\left(\frac{\Delta R}{R_{0}}/\varepsilon \right)$ , was employed to quantify strain sensitivity prior to fabrication of the iontronic sensor. The GF was calculated following previously reported methodologies, and the results are shown in Figure 5e. The GF-strain relationship can be divided into three linear regions, each exhibiting high linear correlation (*R* ^2^ ≈ 0.999), indicating predictable and stable electromechanical behavior over a broad deformation range.

In the low-strain region (0–200%), the hydrogel exhibits a GF of 6.32, enabling sensitive detection of small deformations relevant to epidermal motion. In the intermediate region (201–500%), the GF increases to 10.16 and further rises to 12.08 in the high-strain region (501–650%). This strain-dependent enhancement of sensitivity is attributed to progressive elongation and partial disruption of ionic conduction pathways, which amplify resistance variation at larger deformations. Similar multiregime GF behavior has been reported for advanced ionic conductive hydrogels; however, maintaining high linearity across such an extended strain window remains challenging in many systems.^20,46^

The maximum GF of 12.08 achieved at large-strain levels is within the upper range reported for stretchable ionic hydrogels that do not rely on conductive fillers or complex multinetwork architectures.^4,58,59^ For instance, several state-of-the-art systems exhibit GF values typically below 10 or only within narrow strain windows, often requiring complex multinetwork architectures or filler reinforcement.^40,57,43^ Importantly, this sensitivity is maintained across a wide strain window (up to 650%), highlighting the effectiveness of the hydrophobically associated and hydrogen-bonded architecture in balancing mechanical compliance and electrical responsiveness. As shown in Figure 5f, the hydrogel reliably tracks both slow and rapid deformation modes, indicating a minimal signal lag and stable dynamic response.

Overall, the combination of a wide sensing range, high linearity, rapid response, and robust cyclic stability demonstrates that the synergistic integration of HpA, hydrogen bonding, and ionic conduction provides an effective and structurally simple strategy for developing durable and high-performance wearable strain sensors.

### Application of P­(SMA-*co*-AAm)/Gelatin Hydrogel Sensor for Human Motion Monitoring

As demonstrated in the previous sections, the engineered GT_0.15_ hydrogel exhibits a balanced combination of properties required for wearable strain sensors, including high sensitivity, a wide detectable strain range, rapid response and recovery, and excellent cyclic stability. Such an integrated performance profile is essential for practical human motion monitoring, where sensors must reliably capture both large-amplitude movements and subtle physiological deformations under repeated mechanical loading.

To evaluate the feasibility of the GT_0.15_ hydrogel as an epidermal strain sensor, hydrogel strips were prepared with copper electrodes attached to both ends and connected to an electrochemical workstation (Autolab) via conductive wires. The sensor was directly affixed to selected skin regions of a volunteer by using medical adhesive tape, enabling real-time monitoring of resistance variations induced by body movements. This straightforward configuration, without complex encapsulation layers or auxiliary structural supports, highlights the conformability and mechanical compatibility of the hydrogel with the human skin.

As shown in Figure 6a–g, the hydrogel sensor clearly and reproducibly detects a broad spectrum of human motions, including wrist bending, finger flexion, facial expressions, bilateral leg kicking, speech, and handwriting. Large-amplitude movements, such as wrist bending and leg motion, generate pronounced and stable resistance variations, whereas subtle physiological activities, such as facial muscle movement, speech-related vibrations, and writing, produce well-defined and distinguishable electrical responses. These results confirm the wide dynamic sensing window and stable electromechanical coupling of the hydrogel under diverse deformation modes.

**Figure 6:**
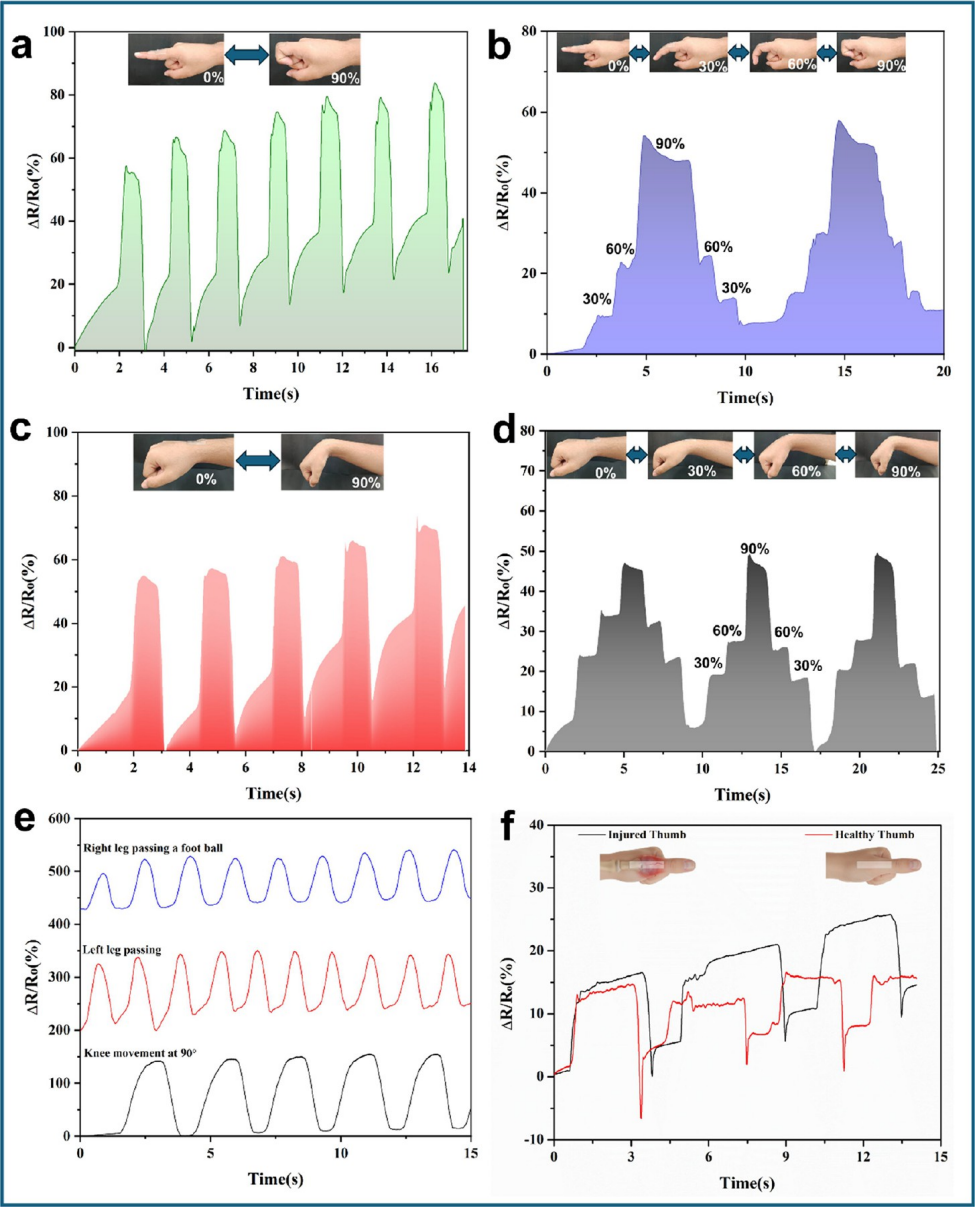
Human motion monitoring using the hydrogel strain sensor under large joint deformations. (a, b) Electrical response to finger bending at single and multiple bending angles. (c, d) Wrist flexion and extension at fixed and varied angles. (e) Knee bending motions during simulated kicking and controlled joint flexion. (f) Comparison of electrical responses from healthy and injured thumb bending, demonstrating sensitivity to joint condition.

Compared with recently reported hydrogel-based strain sensors, many systems exhibit high sensitivity only within limited strain ranges or show reduced accuracy when detecting subtle deformations under large or repeated strains.^46,19^ Other approaches achieve fine-motion resolution but often rely on multilayer architectures, conductive fillers, or encapsulation strategies that increase fabrication complexity or compromise transparency and mechanical compliance.^40,57^ Alternatively, the present system integrates mechanical robustness, transparency, and strain sensitivity within a single physically cross-linked ionic network, enabling reliable detection of both macro- and microscale human motions using a structurally simple device configuration.

Within this framework, the GT_0.15_ hydrogel achieves a wide strain detection range (up to 650%), a maximum gauge factor of 12.08, excellent fatigue resistance, and a fast electromechanical response, all within a single dynamically cross-linked network. The stable and reproducible electrical signals recorded during activities such as speech and handwriting further indicate suitability of this hydrogel for applications requiring precise detection of subtle physiological motions alongside large deformations. These characteristics suggest potential applicability in areas such as human–machine interfaces, gesture recognition, and real-time physiological monitoring, which are increasingly relevant for next-generation wearable electronics.^20,43^

A comparative analysis with recently reported hydrogel-based strain sensors (Table 2) indicates that the present system (i.e., P­(SMA-*co*-AAm)/gelatin network) achieves a competitive balance among strain range, sensitivity, mechanical durability, and device simplicity, without relying on complex multilayer architectures or conductive fillers.

**Table 2:** Benchmarking of the P­(SMA-co-AAm)/gelatin etwork against reported strain-sensing ydrogels^a^,^b^

**hydrogel system**	**stretchability (%)**	**sensitivity (GF)**	**transparency (%)**	**response/recovery time (ms)**	**ref.**
PVA/SA/PEDOT:PSS	209	0.57 (200%)		100/100	^60^
MC/LiCl/TA@CNCs	663	2.27 (600%)	98	216/227	^34^
PVA–PDA-CNC-MXene	863	4.23 (300%)		√	^61^
PAAm/PEDOT:PSS	1000	1.315 (60%)		330/177	^62^
Tre-Zr-AA	1542	7 (800%)	92	150/180	^63^
(PNIPAM/PAA)/TA/Fe	2638	4.368 (500%)	√	269/282	^64^
(κ-carrageenan/PAA)/([EMIM]Cl)	3000	2.32 (300%)	90	280/350	^65^
P(DEEA-*co*-IBA)/IL	√	4.1 (250%)	90	100/190	^66^
PVA/PAM/TA	1097	6 (500%)		100/120	^67^
		15.4 (1097%)			
(PVA/PAA/Zr^4+^)	2053	3.5 (500%)	97	150/230	^68^
		19 (2000%)			
polyacrylamide/starch/ChCl	2580	3.46 (500%)	81.9	120/150	^69^
		15.36 (2580)			
P(BMA-*co*-LA)/IL	540	2.6 (300%)		90/130	^70^
PSG-Zr^4+^-CNT	632	2 (632%)		70/120	^71^
PAA/PAM/PEI/LiCl/CNT/NCG	1022.4	63.3 (500%)		72/116	^72^
P(SMA-*co*-AAm)/gelatin	2420	10 (500%) 12.08 (650%)	85.9	134/124	*this work*

a Symbols: Blank—not applicable; √—not reported.

b Abbreviations: κ-carrageenan—kappa-carrageenan, LiCl—lithium chloride, MC—methylcellulose, MXene—two-dimensional transition-metal carbide/nitride, PAA—poly­(acrylic acid), PDA—polydopamine, PEDOT:PSS—poly­(3,4-ethylenedioxythiophene):poly­(styrenesulfonate), PNIPAM—poly­(*N*-isopropylacrylamide), PVA—poly­(vinyl alcohol), SA—sodium alginate, TA@CNCs—tannic-acid-coated cellulose nanocrystals, Tre—*d*-(+)-trehalose anhydrous, Zr—zirconium, [EMIM]­Cl—1-ethyl-3-methylimidazolium chloride, IBA—isobornyl acrylate, DEEA—di­(ethylene glycol) ethyl ether acrylate, IL—ionic liquid, BMA—butyl methacrylate, LA—lauryl acrylate, ChCl—choline chloride, PSG—PAA/alginate/gelatin, PEI—polyethylenimine.

The hydrogel sensor was affixed to the knuckle of the index finger (interphalangeal joint) to evaluate its electromechanical response during the finger motion. As shown in Figure 6a and Video S2, repeated bending and relaxation of the finger generated distinct and reproducible changes in the electrical signal. To further assess angular sensitivity, the finger was bent to different predefined angles (Figure 6b). The corresponding electrical response exhibited a clear and monotonic dependence on the bending angle, with resistance increasing progressively as the angle increased and returning to its baseline value upon relaxation. This behavior demonstrates stable signal reversibility and reliable strain-dependent electromechanical coupling under cyclic joint deformations.

The increase in resistance during bending can be attributed to elongation of ionic transport pathways and partial narrowing of conductive channels within the hydrogel network under tensile deformation.^29^ Upon relaxation, the hydrogel recovers its original dimensions, reestablishing the ionic conduction pathways and restoring the baseline resistance. This reversible electromechanical response confirms the effective coupling between mechanical deformation and ionic transport within the dynamically cross-linked network. A similar sensing behavior was observed for the wrist motion. As shown in Figure 6c and Video S3, the hydrogel reliably monitored repeated up-and-down wrist movements at a fixed bending angle, while Figure 6d demonstrates stable and distinguishable resistance variations at multiple predefined angles. These results further validate the ability of the hydrogel sensor to reproducibly track the joint motion with varying amplitudes.

Preliminary tests were also conducted on a volunteer’s thumb presenting a minor injury. As shown in Figure 6f, differences in movement dynamics between the injured and healthy thumb produced distinguishable electrical signal patterns. While these observations are qualitative, they suggest sensitivity of the hydrogel sensor to variations in joint motion characteristics. Additionally, beyond large joint movements, the hydrogel sensor demonstrated the capability to detect subtle physiological signals. When attached to the throat region, speech-related muscle activity generated well-defined resistance fluctuations (Figure 7a). Additionally, placement on the forehead enabled monitoring of frowning-induced skin deformation (Figure 7b) and positioning above the navel allowed real-time recording of abdominal expansion and contraction during breathing (Figure 7c).

**Figure 7:**
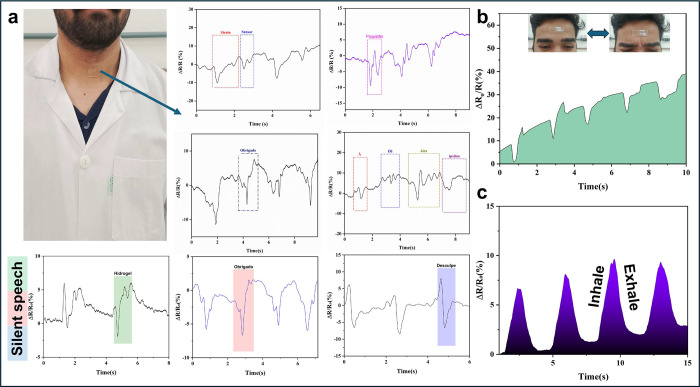
Response of the GT_0.15_ hydrogel sensor to subtle human physiological activities. (a) Electrical response to vocal vibrations when the sensor is attached to the volunteer’s larynx during speech. (b) Electrical response to facial muscle movement during frowning. (c) Electrical response to abdominal expansion and contraction during breathing.

For evaluation of laryngeal motion sensing, the GT_0.15_ hydrogel electrode was affixed to the larynx of a volunteer to detect mechanical vibrations associated with both voiced and silent articulation. During audible pronunciation of the word “Paquistão (Pakistan)”, the sensor generated consistent and repeatable resistance variations, indicating stable detection of speech-related muscle activity and throat vibrations. To further explore signal differentiation capability, individual letters (A, D, J, and Y) were pronounced sequentially. Each articulation produced distinguishable electrical response patterns, suggesting that the hydrogel sensor can resolve subtle differences in laryngeal movement dynamics. Similarly, when different words (e.g., “Obrigado” (thanks) and “strain sensor”) were pronounced repeatedly, the resulting resistance profiles exhibited reproducible and word-dependent temporal features.

Importantly, when the hydrogel was attached to a second volunteer for silent articulation of “Obrigado”, comparable resistance fluctuation patterns were observed relative to the voiced condition. Additional Portuguese words used in silent articulation, including “Hidrogel” (hydrogel) and “Desculpe” (sorry), also produced clearly distinguishable signal profiles. While no advanced signal processing or pattern recognition algorithms were applied in this study, the reproducible and word-dependent electromechanical responses indicate the capability of the hydrogel sensor to detect fine variations in laryngeal muscle activity.

The recorded signals showed good repeatability, with minor variations in peak amplitude primarily associated with differences in the vocal intensity or articulation force. During resting intervals between utterances, the baseline signal remained stable without noticeable drift, confirming a low noise level and a stable electromechanical performance. Repeated pronunciation of the same word generated peaks with consistent temporal features, while amplitude variations reflected differences in the applied mechanical stimulus.

Furthermore, in addition to detecting subtle physiological activities, the hydrogel sensor responds sensitively to localized mechanical stimuli associated with handwriting motions applied directly to its surface. As demonstrated in Figure 8a and Video S4, writing different alphabets (A, B, C, and D) on the hydrogel produces distinct and reproducible electrical response patterns, reflecting differences in stroke sequence, direction, and applied pressure. This investigation was extended to additional letters (E–Z), where consistent and distinguishable signal profiles were observed for repeated writing motions. Although no advanced signal processing or classification algorithms were employed in this study, the reproducible and letter-dependent electromechanical responses indicate the capability of the hydrogel to resolve fine mechanical variations associated with complex motion patterns.

**Figure 8:**
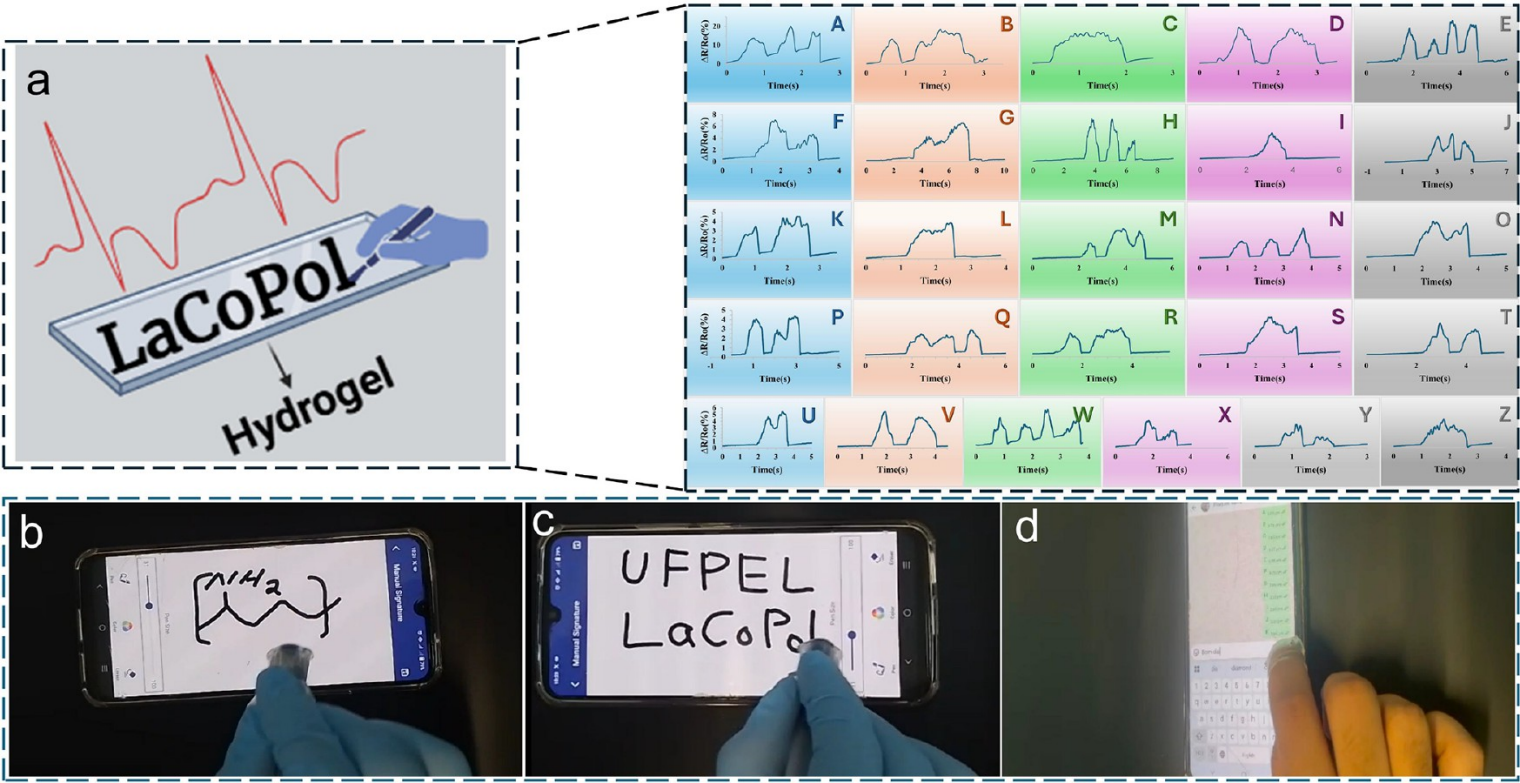
Demonstration of handwriting recognition and touchscreen interaction using the P­(SMA-*co*-AAm)/gelatin hydrogel (GT_0.15_ sample) sensor. (a) Electrical responses generated during handwriting of alphabets from A to Z. (b, c) Drawing of a molecular structure and writing on a smartphone using a hydrogel-based pen. (d) Hydrogel tip attached to a human finger enabling reliable touchscreen operation as an electronic pen.

These findings suggest potential applicability in fine-motion monitoring and interactive human-machine interfaces, particularly in systems where the mechanical input is translated into electrical signals through deformable and transparent soft materials.

In addition to its sensitivity across a wide range of human motions, the hydrogel also presents potential as a functional component in electronic skin (e-skin) systems, owing to its mechanical robustness, stable ionic conductivity, and soft, compliant characteristics. The combination of elasticity, transparency, and strain-dependent electromechanical response makes it suitable for integration into deformable sensing platforms designed to mimic the mechanical behavior of human skin.^73^ Importantly, a smooth and skin-compatible surface is essential to minimizing irritation and mechanical discomfort during prolonged contact with the body. To further evaluate its practical applicability, the ability of the hydrogel strain sensor to interface with capacitive touchscreen devices, which is a relevant functionality for wearable electronics, was investigated.

As shown in Figure 8b,c (and Video S5), the hydrogel enabled effective capacitive touchscreen operation, allowing a user to write multiple alphabets and draw a molecular structure pattern on a mobile device. When fitted onto the index finger of a volunteer, the hydrogel maintained a reliable touchscreen responsiveness. In contrast to many electrically insulating materials that impede capacitive coupling, the ionic conductive nature of the hydrogel allowed for effective signal transmission during finger-based interaction.

To clarify the role of human contact in this process, we conducted control experiments. When the hydrogel was in direct contact with human skin, it functioned as a conductive interface for the touchscreen manipulation. However, in the absence of human–hydrogel contact, no touchscreen response was observed. Further verification was performed by mounting the hydrogel onto a plastic pen and attempting interaction with a touchscreen application without skin contact; under these conditions, the device remained unresponsive. These observations indicate that capacitive coupling between the human body and the touchscreen is necessary to activate the conductive pathway through the hydrogel, highlighting its function as an ionically conductive extension of the finger rather than as an independent stylus. As further illustrated in Figure 8d, the hydrogel demonstrated stable performance across different touch-based tasks, supporting its suitability for integration into interactive wearable platforms.

Overall, the P­(SMA-*co*-AAm)/gelatin hydrogel, particularly the optimized GT_0.15_ formulation, achieves a balanced combination of high stretchability, enhanced fracture stress, broad strain sensitivity, fast response/recovery, and excellent cyclic stability within a single physically cross-linked ionic network. As summarized in Table 2, this performance is competitive with and, in several aspects, comparable to recently reported hydrogel-based strain sensors, while avoiding the use of conductive fillers, rigid nanoreinforcements, or complex multilayer architectures. The ability to simultaneously maintain high optical transparency, mechanical robustness, and strain sensitivity highlights the effectiveness of the dual dynamic cross-linking strategy employed here.

Beyond performance metrics, this study provides new insights into the cooperative role of micelle-mediated HpA and gelatin-induced hydrogen bonding in regulating network homogeneity, energy dissipation behavior, and electromechanical coupling. The results demonstrate that fine control over reversible physical interactions can enable a favorable balance between elasticity and durability without resorting to sacrificial bond rupture or permanent covalent reinforcement. This structure–property relationship contributes to the rational design principles of sustainable, filler-free ionic conductive hydrogels for soft electronics.

It should be noted that the present study primarily focuses on the mechanical and electromechanical characterization under controlled laboratory conditions. Despite the promising performance, some limitations should be considered for practical applications. In particular, key aspects such as long-term environmental stability (e.g., dehydration resistance), temperature tolerance, extended fatigue performance beyond 10^3^ cycles, and in-depth biocompatibility assessments were not systematically investigated in this work. These factors are critical for real-world wearable applications and will be addressed in future studies. Moreover, the integration of the hydrogel into fully packaged, wearable devices remains to be explored. Future developments incorporating advanced signal processing strategies and sensor array configurations may further expand the capabilities of the material toward motion pattern recognition, multipoint sensing, and more complex human-machine interaction systems.

## 4 Conclusions

In summary, a bioreinforced ionic conductive hydrogel was developed through a simple and scalable fabrication strategy that integrates HpA, hydrogen bonding, and ionic conduction within a single physically cross-linked network. The incorporation of gelatin, a renewable and biocompatible macromolecule, provides a sustainable approach to enhance mechanical robustness while preserving optical transparency and processing simplicity. In contrast to many high-performance conductive hydrogels that rely on conductive fillers, rigid nanoreinforcement, or complex multinetwork architectures, the present system achieves a balanced combination of mechanical, optical, and electrical performance using low-cost and widely available components. The rational network design, based on micelle-mediated hydrophobic domains cooperatively interacting with reversible hydrogen bonds, enables efficient strain-dependent energy dissipation and rapid elastic recovery. As a result, the optimized hydrogel (GT_0.15_ sample) exhibits ultrahigh stretchability (2420%), enhanced fracture stress (0.31 MPa), high optical transparency (85.9%), and stable ionic conductivity, supporting reliable strain sensing across a broad deformation range (0.5–650%). The material further demonstrates high sensitivity (maximum gauge factor of 12.08), fast response/recovery, and excellent cyclic durability, confirming stable electromechanical performance under repeated deformation.

Beyond performance metrics, this study provides insight into how controlled supramolecular interactions in a bioreinforced polymer network can regulate structural homogeneity, mechanical resilience, and ionic transport without requiring sacrificial covalent bonds or conductive fillers. This structure–property understanding contributes to the rational design of transparent and mechanically robust ionic hydrogels for soft electronics. Overall, this work establishes a scalable and sustainability-oriented material-engineering strategy for multifunctional soft conductive hydrogels, advancing the development of practical and mechanically resilient sensing platforms for wearable and interactive electronic systems.

## Supplementary Material













## Notes

Images of the electrochemical cell equipped with two circular stainless-steel electrodes used as current collectors; electrochemical impedance spectroscopy (EIS) curves and the corresponding calculated ionic conductivity of the hydrogels are also provided; manual strain sensing of the GT0.15 sample demonstrated using a customized experimental setup.(PDF)
Gradual decrease in LED brightness upon progressive stretching of the hydrogel, indicating an increase in electrical resistance during deformation (MP4)
Reproducible current variations during repeated bending and unbending of a finger, confirming a stable electromechanical response (MP4)
Capability of the hydrogel sensor to monitor repetitive up-and-down wrist movements at a fixed bending angle (MP4)
Distinct and recognizable electrical response patterns generated during handwriting of different letters (A, B, C, and D) (MP4)
Application of the hydrogel sensor for capacitive touchscreen operation, enabling the user to write multiple letters and draw a molecular structure on a mobile device (MP4)
