# Supplementary material for: Engineering Dynamic Hydrophobic Domains in Bioreinforced Ionic Hydrogels for Robust and Transparent Soft Electronics
Source: Langmuir. 2026 Jun 9;42(24):17455–71. doi: 10.1021/acs.langmuir.6c01278 (PMC13296479; doi:10.1021/acs.langmuir.6c01278)
Supplement: Supplementary file 1 [file la6c01278_si_001.pdf]

Supplementary material for

Engineering dynamic hydrophobic domains in bio-reinforced ionic hydrogels for robust and transparent soft electronics

Ijaz Ali [a], Mansoor Khan [b], Eliaquim B. P. Sena [a], André R. Fajardo [a]*

[a] Laboratório de Tecnologia e Desenvolvimento de Compósitos e Materiais Poliméricos (LaCoPol), Federal University of Pelotas, 96010-900, Pelotas-RS, Brazil.
[b] School of Materials Science and Engineering, Shanghai University, Shanghai 200444, China.

*Corresponding author: Prof. Dr. André R Fajardo (e-mail: andre.fajardo@ufpel.edu.br)

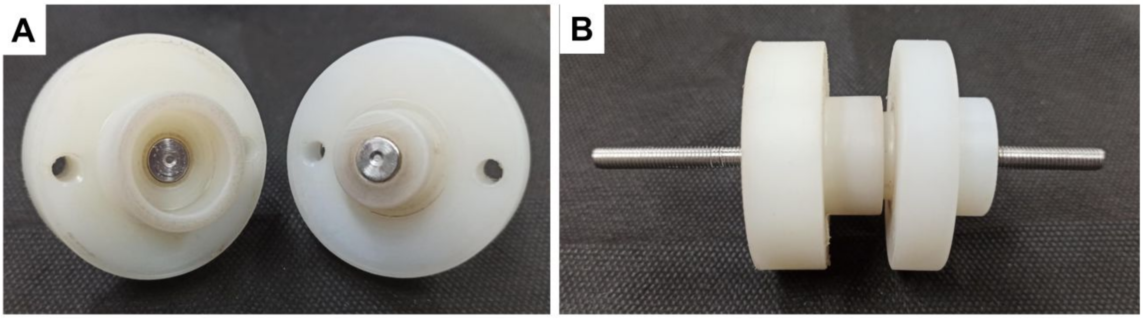

Figure S1. Electrochemical cell containing two circular stainless-steel electrodes (A – separated and B – together) as current collectors.

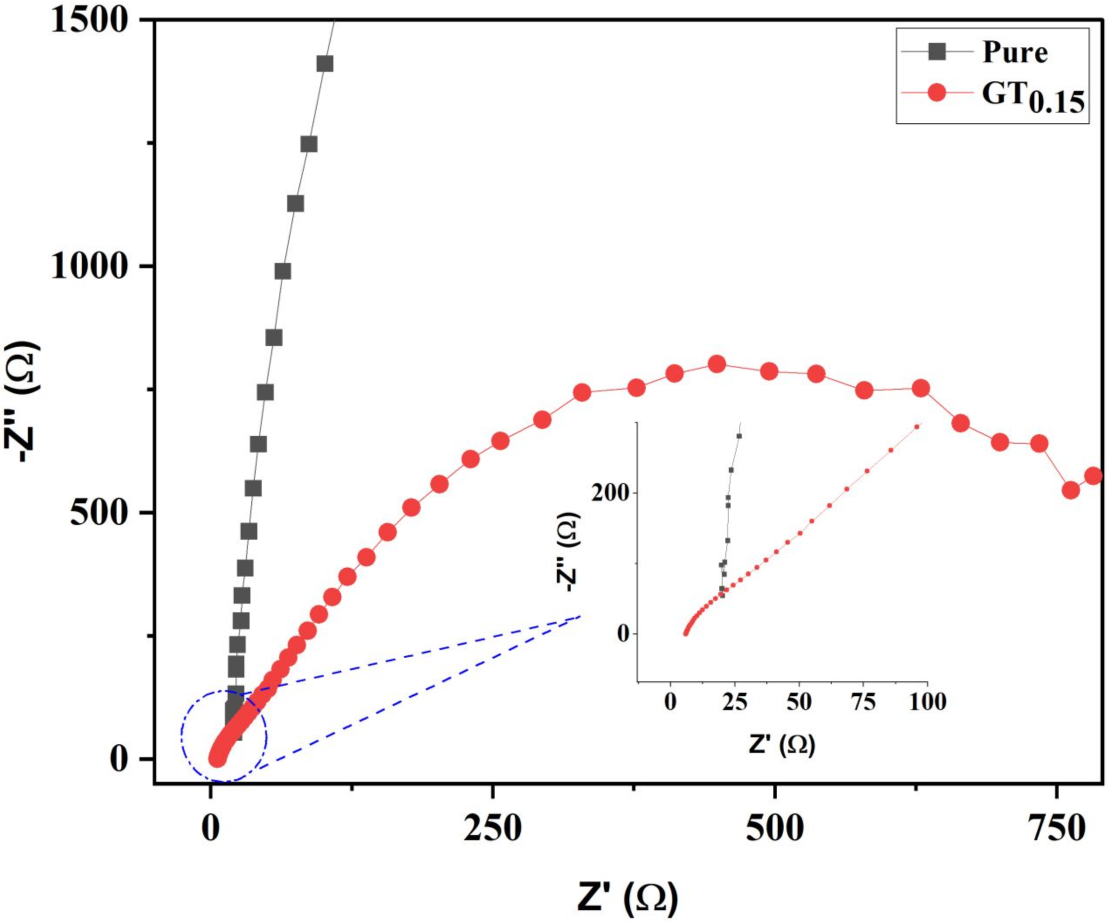

Figure S2. Electrochemical impedance spectroscopy (EIS) curves for Pure and $GT_{0.15}$ samples.

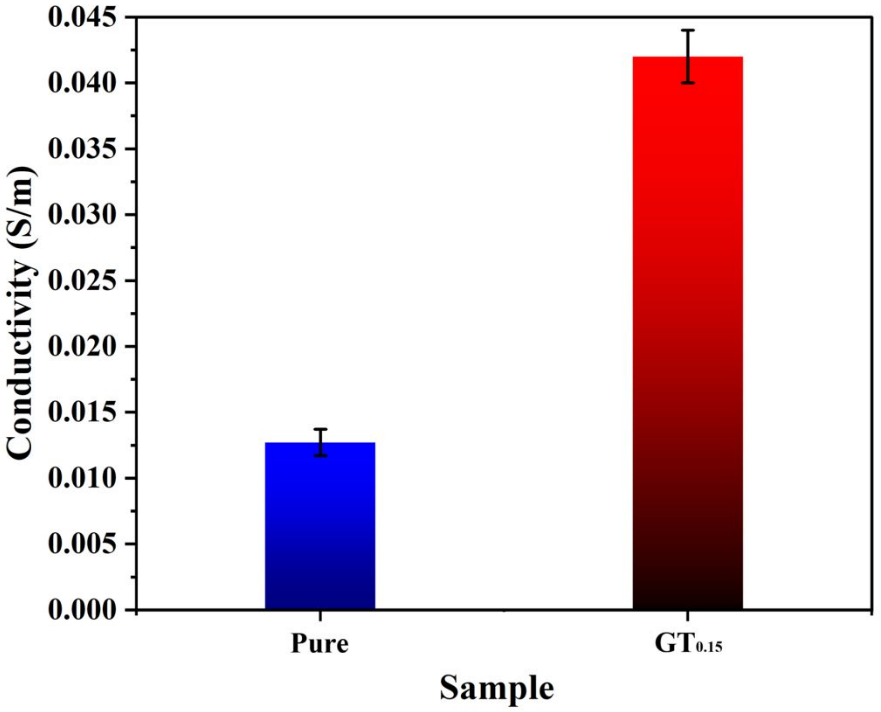

Figure S3. Calculated graph of the ionic conductivity for Pure and $GT_{0.15}$ samples.

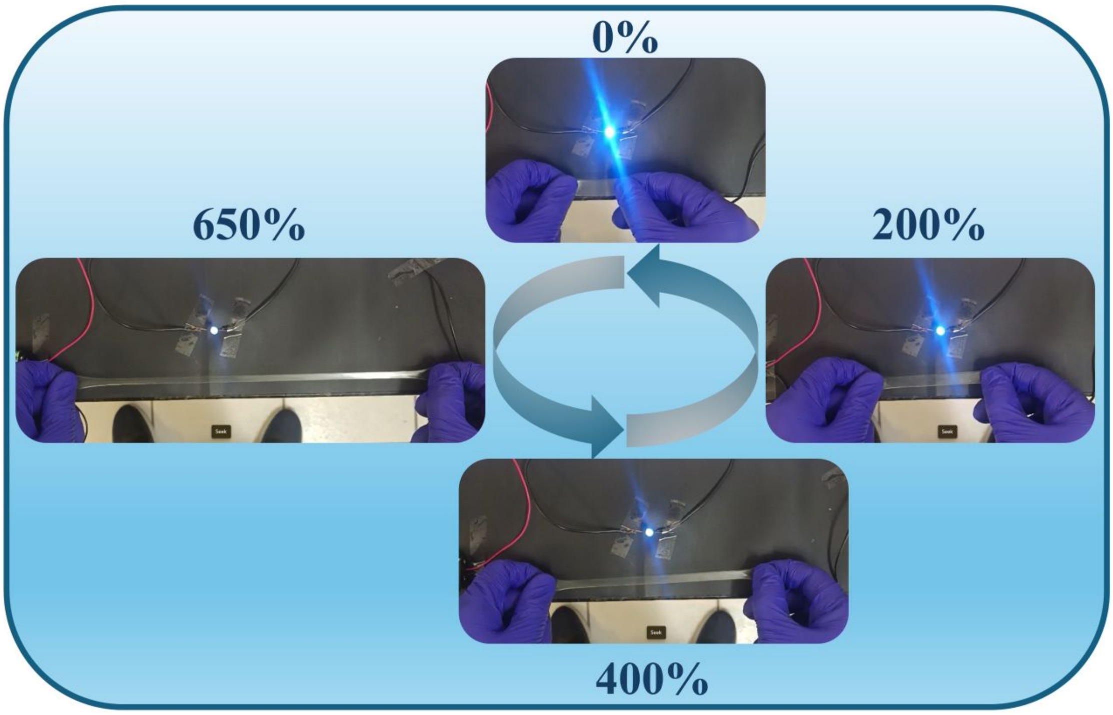

Figure S4. Manual strain sensing of the $GT_{0.15}$ through a customized setup.
